# A transcriptome sequence dataset characterizing eggs, nymphs and adults of *Oxycarenus hyalinipennis*, the cotton seed bug

**DOI:** 10.1016/j.dib.2026.112532

**Published:** 2026-02-05

**Authors:** Sam D. Heraghty, Aijun Zhang, Daniel Kuhar, Dawn E. Gundersen-Rindal, Michael E. Sparks

**Affiliations:** Invasive Insect Biocontrol and Behavior Laboratory, USDA-ARS, Beltsville, MD 20705, USA

**Keywords:** Transcriptomics, Gene expression, Invasive insects, Agricultural pests

## Abstract

The cotton seed bug, *Oxycarenus hyalinipennis,* is an agricultural pest that has recently been detected in the United States and has the potential to cause extensive economic damage to the cotton production industry. Currently, there are no transcriptomic resources for this species. The data reported here will serve to help guide future efforts to create additional reference resources as well as facilitate the development of population control strategies. These data could also be of use towards identifying protein coding genes in a cotton seed bug genome assembly. A total of 13,384 differentially expressed genes was identified, which collectively encoded 40,871 distinct transcripts, of which 18,842 could be annotated with a reference protein in the NCBI NR database, 13,233 with Pfam protein families and 8,089 with GO Gene Ontology terms. These transcripts could, for example, be targeted for future functional genomics work.

Specifications Table

**Table:** 

Subject	Biology
Specific subject area	Whole-insect transcriptomics data from the egg, 2^nd^ instar, 4^th^ instar, adult male, and adult female life stages of the cotton seed bug (*Oxycarenus hyalinipennis*)
Type of data	Illumina PE150 RNA-Seq data, reads trimmed per quality information (FASTQ format).
Data collection	Three whole-insect biological replicates apiece were prepared for each of 2^nd^ and 4^th^ nymphal instars, and ten-day-old, unmated male and female adults. Additionally, one replicate of the egg stage was prepared. *Oxycarenus hyalinipennis* insects were reared in a culture maintained at the Beltsville Agricultural Research Center. Three egg masses were combined for the egg replicate; 100 and 50 individuals were pooled per 2^nd^ and 4^th^ instar replicate, respectively; and five individuals for adult male and female replicates. Libraries were prepared and sequenced on an Illumina NextSeq2000 at the Georgia Genomics and Bioinformatics Core, which also performed quality-based read trimming.
Data source location	Biological sequence data are stored and made publicly available at the NCBI in Bethesda, Maryland, USA
Data accessibility	Repository name: National Library of Medicine - National Center for Biotechnology Information – BioProject Division Data identification number: BioProject accession number PRJNA1151619 Direct URL to data: https://www.ncbi.nlm.nih.gov/bioproject/?term=PRJNA1151619 Data can be accessed directly via the NCBI website using built-in functions or via the NCBI Datasets toolkit (https://github.com/ncbi/datasets). Alternatively, the transcriptome assembly can be accessed at the NCBI Nucleotide division under accession GKYF00000000 (https://www.ncbi.nlm.nih.gov/nuccore/GKYF00000000).
Related research article	None

## 1 Value of the Data

• This dataset provides the first set of transcriptomic resources for this nuisance insect, providing both a *de novo* transcriptome assembly as well as life stage-specific expression data. Such resources will be critical in further understanding the biology of this rapidly emerging pest.
• Due to both the robust replication in the experimental design used to generate this dataset, as well as the fact that sequencing was performed simultaneously on a single instrument, this dataset can reliably identify genes that are differentially expressed across life stages and sexes. A limited quantitative analysis is described and provides a listing of differentially expressed genes to the research community, which could be useful for informing future functional genomics studies, gene family phylogenetic analyses, and so forth. Characterizing life stage- and sex-specific patterns may provide useful insight into the development of novel population control measures.
• The data produced here will be generally useful in understanding evolutionary patterns across insects, especially within the order Hemiptera. For instance, this dataset could be combined with other publicly available data to address questions related to gene family evolution.
• This dataset will be useful to the research community in future efforts to annotate a reference genome for this species and/or as a resource for phylogenetic analysis of gene families of interest.

## 2 Background

The cotton seed bug, *Oxycarenus hyalinipennis* Costa (Hemiptera: Lygaeidae), is a widespread invasive pest species with a broad breadth of host plants including those in the order Malvales, which includes cotton (*Gossypium* spp.). Native to Africa, it has spread globally and is now found in Asia, Europe, South America and the Caribbean [1]. Cotton seed bug was previously detected in Florida in 2010 but was subsequently eradicated [2]. However, cotton seed bug has recently been detected in southern California [3]. This pest is likely to invade the southern U.S., a major producer of cotton [4], either from spread of the population in the western U.S. or from a novel invasion from populations in the Caribbean. There are currently no transcriptomic or genomic resources for this species. The data described here will be useful in developing new molecular control techniques for this pest as well as contributing towards the development of more expansive genomic resources (e.g., a well-annotated reference genome).

## 3 Data Description

Unassembled, quality-trimmed Illumina RNA-Seq reads and a global transcriptome assembly are available at NCBI under BioProject accession PRJNA1151619 [5]. Gene expression and transcriptome annotation, supporting software, data quality control reports, predicted protein and mRNA sequences, clustered and/or filtered variants of the assembly and a supplementary table are available in the supplementary materials, which are described below and made available from the Center for Open Science’s data sharing platform, the Open Science Framework, at https://doi.org/10.17605/OSF.IO/DS8CJ. Please see the “0_Supplement_Description.docx” file provided therein for a detailed description of the supplementary materials.

## 4 Experimental Design, Materials and Methods

Cotton seed bugs were originally obtained from primrose street trees, *Lagunaria patersonia*, infested with *O. hyalinipennis* in Irvine, Orange County, CA on 24 October 2021 and then reared in containment at the USDA-ARS Invasive Insect Biocontrol and Behavior Laboratory in Beltsville, MD for approximately nine months. Briefly, adults were reared in transparent plastic rectangular containers fitted with nylon screens and containing dry cotton seeds, organic green beans and a water source as described by Saveer et al. [3]. Adults were aged to ten days after final instar molting. Each life stage was reared in separate containers and maintained in a controlled environment chamber at 26°C ± 1°C and 75% relative humidity, and a 16 h light/8 h dark cycle.

Three biological replicates (bioreps) apiece for each of 2^nd^ and 4^th^ nymphal instars, as well as female and male adults, were prepared utilizing the RNAqueous Total RNA Isolation kit (ThermoFisher, Waltham, MA). Owing to difficulties in obtaining sufficient biological material, only a single biorep of the egg stage was prepared. Due to size differences between eggs, instars and adults, three egg masses were pooled for its biorep, 100 individuals were pooled per biorep for 2^nd^ instar nymphs, 50 per biorep for 4^th^ instars, and five individuals per biorep for adult males and females. Individuals were collected from cotton bolls and placed directly into RNAlater (ThermoFisher, Waltham, MA) using forceps and subsequently stored at -80°C until RNA extraction. For RNA extraction, insects were placed in 2mL matrix A lysing tubes and pulverized using MP BioMedicals’ (Solon, OH) Fastprep 24 for 60 seconds in 300uL lysis buffer. RNA was extracted per the RNAqueous protocol and further purified using Turbo DNAse (ThermoFisher, Waltham, MA) to remove any DNA contamination. RNA concentration was determined using Promega’s (Madison, WI) Quantas fluorometer. Samples were aliquoted and sent to the University of Georgia’s Georgia Genomics and Bioinformatics Core (Athens, GA) for Illumina PE150 RNA-Seq sequencing on a NextSeq2000 instrument. Read quality was assessed using FastQC (v0.12.1; [6]); please see “fastqc_html_reports.tar.gz” in the supplementary materials for quality reports).

Total RNA-Seq sequencing volumes are presented in Table 1. Reads from all samples were globally pooled and normalized using the *in silico* normalization tool from the Trinity package (v2.13.2; [7]). Trinity was also used to produce a de novo assembly of the normalized reads, the results of which were used for downstream quantitative and qualitative analyses. Quality of the assembly was assessed using BUSCO v6.0.0 [8] with the hemiptera_odb12 dataset [9].

**Table 1:** Raw sequencing volumes achieved for each developmental stage and/or sex characterized, per biological replicate (biorep). A target length of 150bp was used for each paired read. Reads were quality trimmed in advance by the sequencing vendor. Note that only a single biorep is available for the Egg stage.

	Egg Stage		
	*biorep 1*	*biorep 2*	*biorep 3*
read pairs	51,079,074	*not applicable*	*not applicable*
bases	15,425,880,348	*not applicable*	*not applicable*

	2^nd^ Instar Nymphs		
	*biorep 1*	*biorep 2*	*biorep 3*
read pairs	40,487,424	35,950,857	38,334,372
bases	12,227,202,048	10,857,158,814	11,576,980,344

	4^th^ Instar Nymphs		
	*biorep 1*	*biorep 2*	*biorep 3*
read pairs	29,273,949	34,042,569	38,789,872
bases	8,840,732,598	10,280,855,838	11,714,541,344

	Male Adults		
	*biorep 1*	*biorep 2*	*biorep 3*
read pairs	38,998,199	38,826,019	32,178,615
bases	11,777,456,098	11,725,457,738	9,717,941,730

	Female Adults		
	*biorep 1*	*biorep 2*	*biorep 3*
read pairs	35,508,779	34,265,719	33,381,413
bases	10,723,651,258	10,348,247,138	10,081,186,726

DESeq2 (v1.49.2; [10]), in conjunction with the salmon mapping tool (v1.9.0; [11]), was used to identify differentially expressed genes in seven comparisons: 2^nd^ vs 4^th^ nymphal instars, 2^nd^ instars vs female adults, 2^nd^ instars vs male adults, 4^th^ instars vs female adults, 4^th^ instars vs male adults, female vs male adults, and nymphs (i.e., 2^nd^ and 4^th^ instars) vs adults (female and male adults). (Identifying differentially expressed genes from egg data was not possible, as only one biological replicate was available.) To obtain gene- and transcript-level expression estimates, the RSEM expression estimation method (v1.3.3; [12]), using read alignment results produced by bowtie 2 (v2.3.4.1; [13]), was invoked via Trinity’s align_and_estimate_abundance utility. Expression measures were conveyed using the Transcripts per Million unit (TPM; [14]).

Assembled transcripts were compared with the NCBI NR protein database (accessed on 19 February 2025) using the BLASTx-like mode of DIAMOND (v2.0.4; [15]) with default parameters. For each assembled transcript, a longest open reading frame (ORF) was found after translating in six frames using the transeq program from EMBOSS (v6.6.0.0; [16]). Longest ORFs were then compared with the Pfam database (version of 5 December 2024; [17]) using the hmmsearch program from HMMer (v3.3.4; [18]) with default parameters (i.e., an E-value inclusion threshold of 0.01). Associated GO terms for protein family matches were identified using the pfam2go table (v2024/04/08; [19]).

RNA-Seq reads were assembled into a global transcriptome of 685,930 mRNA pseudomolecules consisting of 442,270,857 assembled bases in total. Of these, 116,403 exhibited a positive match in the NCBI NR database, 65,364 could be annotated with one or more Pfam families and 38,260 with one or more GO terms. The transcriptome had a BUSCO score of 96.0% (19.8% complete and single copy, 76.2% complete and duplicated, 3.2% fragmented and 0.8% missing). The high duplication rate in this unclustered, unfiltered de novo transcriptome assembly likely stems from utilizing multiple diploid, not-highly-inbred individuals in the various samples used to construct the dataset, which allowed for a greater representation of genetic variation than would be observed if using explicitly inbred lines maintained over many generations in a laboratory culture. Experiments with clustering using CD-HIT (v4.8.1; [20]) noticeably improved BUSCO scores, whereas filtering out what were presumably non-host transcripts had little effect (see Supplemental Table 1). Expression levels for every transcript from the global cotton seed bug assembly, expressed in units of Transcripts per Million, are provided in the supplemental file, “CSB_allTcts_TPMs.xlsx”; this file also presents transcript-level Pfam and GO annotations, as well as comprehensive functional profiles of the overall transcriptome in terms of Pfam families and GO terms (for each of the Gene Ontology’s biological process, cellular component and molecular function aspects).

A total of 13,384 differentially expressed genes (DEGs) was identified, to which was attributed a total of 40,871 associated transcripts (due to genes encoding one or more transcripts). Among DEG-associated transcripts, 18,842 could be annotated with an NR reference protein and 13,233 with Pfam terms (of those, 8,089 had associated GO terms). Gene-level expression results for genes observed to be differentially expressed in any of the comparisons are provided on the worksheet labeled “CSB_contrasts_with_TPM” in the supplemental file, “CSB_DE_genes-and-tcts.xlsx”. Transcript-level expression results for DEG-associated isoforms are provided on the “DEG-assoc_tcts” worksheet of this supplemental file, also.

**Fig. 1:**
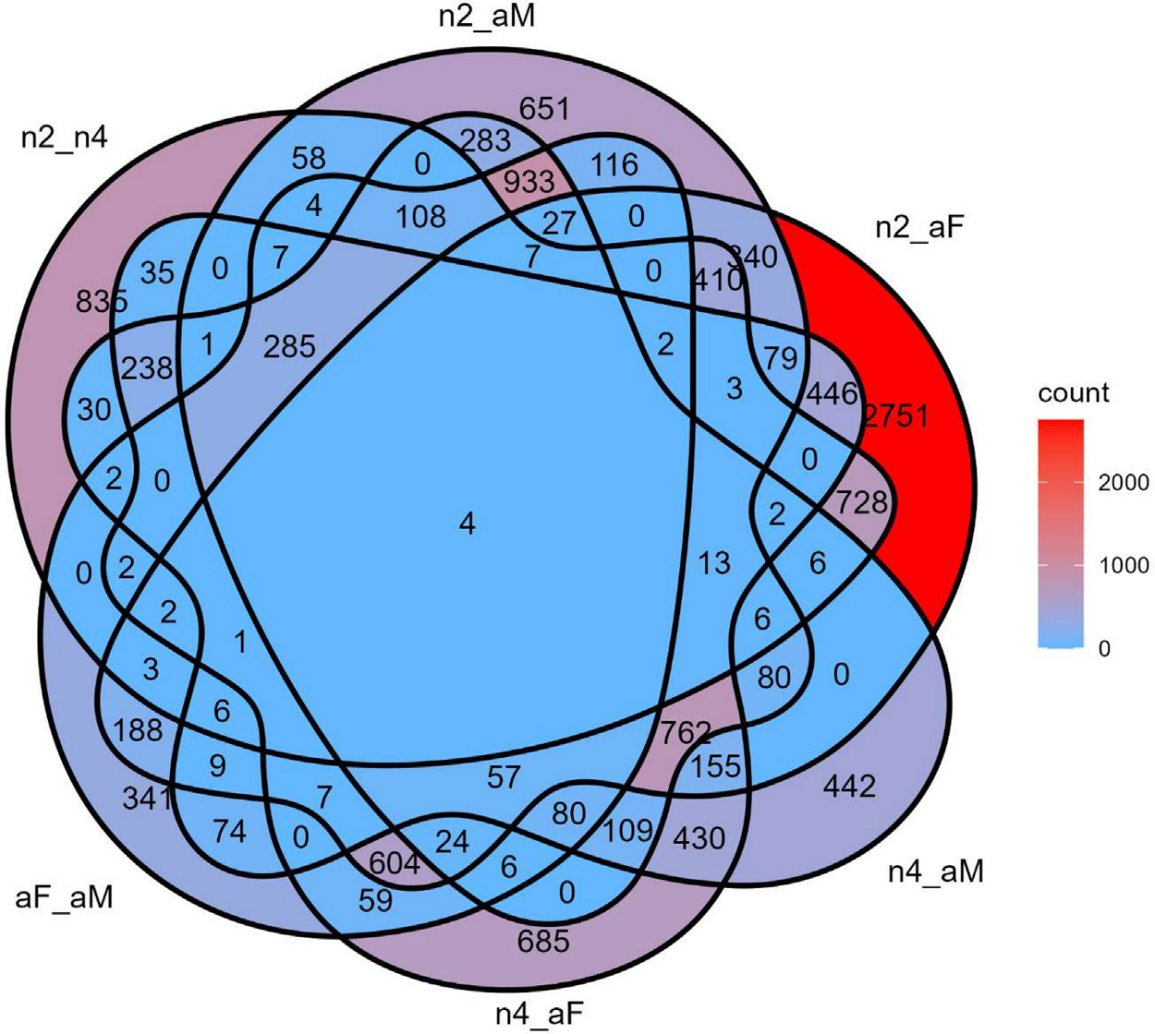
A Venn diagram depicting the distribution of shared and unique differentially expressed genes across all six comparisons. Genes are included regardless of whether they are up- or down-regulated in a particular context. The labels represent the following comparisons: “n2_n4” ∼ 2^nd^ instar versus 4^th^ instar, “n2_aM” ∼ 2^nd^ instar versus adult male, “n2_aF” ∼ 2^nd^ instar versus adult female, “n4_aM” ∼ 4^th^ instar versus adult male, “n4_aF” ∼ 4^th^ instar versus adult female, and “aF_aM” ∼ adult female versus adult male.

The DEG identification method yielded the following comparison-specific DEG tallies from a set of (see “supporting_scripts_CSB.tar.gz” in supplementary materials for detailed results): 2^nd^ vs 4^th^ = 2,798 total (2,040 up, 758 down); 2^nd^ vs female = 6,722 total (4,325 up, 2,397 down); 2^nd^ vs male = 4,455 total (1,392 up, 3,063 down); 4^th^ vs female = 4,098 total (2,661 up, 1,437 down); 4^th^ vs male = 4,157 total (1,672 up, 2,485 down); female vs male = 2,958 total (1,081 up, 1,877 down); and nymph vs adult = 4,384 total (2,366 up, 2,018 down). A Venn diagram summarizing DEG counts across treatment groups was generated using the ggVenDiagram R package (v1.5.2; [21]); see Fig. 1.

**Fig. 2:**
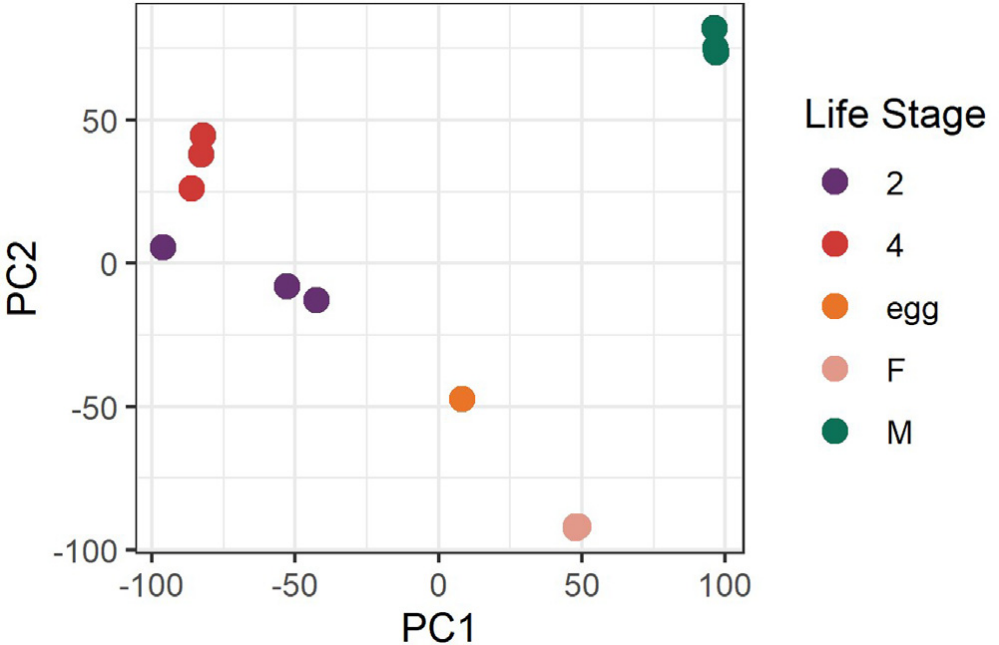
Principal component analysis of RNA-Seq data. The labels represent the following life stages: “2” ∼ 2^nd^ instar, three points; “4” ∼ 4^th^ instar, three points; “egg” ∼ egg stage, one point; “F” ∼ adult female, three points; and “M” ∼ adult male, three points. All three points from the adult female sample are very nearly superimposed on one another, as is also the case for two of the adult male sample points.

The assembled transcriptome contained some transcripts with hits to various fungal taxa (see the “is_fungal?” columns in the supplemental files, “CSB_allTcts_TPMs.xlsx” and “CSB_DE_genes-and-tcts.xlsx”). To better describe this, all 13 biological replicates were independently assembled and annotated, and then assessed for the total number of assembled transcripts exhibiting a match to NCBI NR proteins of fungal origin per the DIAMOND aligner (see Table 2). Samples 2a and 2b had the highest number of matches and samples 4a, 4b, 4c and Ma had somewhat elevated counts; a certain degree of fungal representation was observed among all samples. A principal component analysis, prepared using utilities in the DESeq2 package, similarly demonstrated that samples 2a and 2b were somewhat distinctive from sample 2c (see Fig. 2).

**Table 2:** Transcripts of fungal origin among sample-specific transcriptome assemblies. In the “Sample” column, ‘a’, ‘b’ and ‘c’ indexes the three biological replicates (bioreps) that were sequenced for 2^nd^ instar nymphs (‘2’), 4^th^ instar nymphs (‘4’), adult females (‘F’) and adult males (‘M’); only one biorep was possible from egg (‘Egg’) material. The “Transcripts” column indicates the number of unique transcripts assembled from that respective sample’s read data, “*Aspergillus*” and “*Penicillium*” indicate the number of transcripts matching to reference proteins from that specified genus, “Other” refers to transcripts matching to any other fungal genera, “TotFun” is the total number of transcripts originating from fungi, and “Percentage” gives the percentage of fungal transcripts relative to overall transcripts.

Sample	Transcripts	*Aspergillus*	*Penicillium*	Other	TotFun	Percentage (%)
Egg	174,195	8	6	20	34	0.02
2a	237,207	14,768	2,238	98	17,104	7.21
2b	235,066	12,083	739	7	12,829	5.46
2c	180,721	56	4	19	79	0.04
4a	133,328	18	1	2,186	2,205	1.65
4b	156,975	66	0	2,599	2,665	1.70
4c	162,960	33	2	2,880	2,915	1.79
Fa	116,667	457	34	6	497	0.43
Fb	112,294	197	14	1	212	0.19
Fc	102,977	4	3	0	7	0.01
Ma	150,630	1,245	399	545	2,189	1.45
Mb	146,211	857	289	20	1,166	0.80
Mc	126,701	425	296	12	733	0.58

The biological origins of these fungal reads remain unclear, and as such they present an intriguing opportunity for future research in insect-microbe interactions. A possible hypothesis to test is that they were present due to an undetermined number of nymphal individuals collected from cotton bolls harboring fungi on their exoskeletons, transiently acquired from plant material and present in a manner that was non-endophytic, non-endosymbiotic and non-infectious. (If so, this behavioral capacity for cotton seed bugs to mechanically vector plant pathogens among cotton host plants also underscores the importance of controlling their populations for efficacious plant protection.) The fungal data could also be used to guide research into appropriating fungi for biocontrol purposes, as has been done in other arthropods [22]. The availability of an *O. hyalinipennis* genome assembly, currently in preparation by the authors, could be used to help filter host reads from non-host. In the meanwhile, end users of these RNA-Seq data should note the presence of these fungal reads within the dataset. Overall, these transcriptomic resources will be useful in designing novel approaches to controlling this pest species [23].

## Limitations

The samples used to produce this dataset each consisted of multiple diploid, not-highly-inbred individuals, and are therefore likely to contain greater levels of genetic heterozygosity than could have been achieved after extensive inbreeding in a laboratory culture over numerous generations. Only a single biological replicate of the egg life stage was available, which was used in transcriptome assembly but was not included in differential expression analyses.

## Ethics Statement

The authors have read and followed the ethical requirements for publication in *Data in Brief* and confirm that the current work does not involve human subjects, animal experiments, or any data collected from social media platforms.

## CRediT authorship contribution statement

**Sam D. Heraghty:** Conceptualization, Methodology, Formal analysis, Data curation, Writing – original draft. **Aijun Zhang:** Conceptualization, Resources, Investigation. **Daniel Kuhar:** Resources, Investigation. **Dawn E. Gundersen-Rindal:** Conceptualization, Resources, Investigation, Supervision, Writing – original draft. **Michael E. Sparks:** Conceptualization, Methodology, Formal analysis, Data curation, Writing – original draft.

## Data Availability

National Library of Medicine - National Center for Biotechnology Information – BioProject Divisioncotton seed bug transcriptome (Original data).

## Appendix Supplementary materials

Image, application 1
