# Supplementary material for: A transcriptome sequence dataset characterizing eggs, nymphs and adults of Oxycarenus hyalinipennis, the cotton seed bug
Source: Data Brief. 2026 Feb 5;65:112532. doi: 10.1016/j.dib.2026.112532 (PMC12915258; doi:10.1016/j.dib.2026.112532)
Supplement: Supplementary file 1 [file mmc1.zip › 0_Supplement_Description.docx]

# Supplementary Materials for

A transcriptome sequence dataset characterizing eggs, nymphs and adults of *Oxycarenus hyalinipennis*, the cotton seed bug

Sam D. Heraghty^1^, Aijun Zhang^1^, Daniel Kuhar^1^, Dawn E. Gundersen-Rindal^1^, Michael E. Sparks^1,2^

^1^ Invasive Insect Biocontrol and Behavior Laboratory, USDA-ARS, Beltsville, MD 20705, USA

^2^ michael.sparks2@usda.gov

Description of all supplementary files:

- “supporting_scripts_CSB.tar.gz” – contains in-house scripts and commands used in the construction and analysis of the presented dataset.
- “CSB_allTcts_TPM.xlsx” – spreadsheet containing the expression levels (units are TPM) and top NR hits for all transcripts in the assembled transcriptome; also included are Pfam and GO annotations, both for independent transcripts and for the overall transcriptome.
- “CSB_DE-gene-and-tcts.xlsx” – spreadsheet containing gene- and isoform-level expression (units are TPM) for differentially expressed genes (DEGs) and DEG-associated transcript isoforms, respectively.
- “fastqc_html_reports.tar.gz” – HTML-formatted FastQC reports for sequencing reads, where filenames indicate the sample and read direction (i.e., forward or reverse).
- “sequence-resources/reduced-assemblies/” – directory containing clustered and/or filtered versions of the assembly, as described in the “0README.txt” file therein and in Supplemental Table 1 and commentary below.
- “sequence-resources/CSB_*.txt.gz” – Assembled transcripts and the longest open reading frame observed in each mRNA pseudomolecule.
- “Supplemental_Table_1.docx” – supplementary table, with legend.
