# Supplementary material for: A transcriptome sequence dataset characterizing eggs, nymphs and adults of Oxycarenus hyalinipennis, the cotton seed bug
Source: Data Brief. 2026 Feb 5;65:112532. doi: 10.1016/j.dib.2026.112532 (PMC12915258; doi:10.1016/j.dib.2026.112532)
Supplement: Supplementary file 1 [file mmc1.zip › Supplemental_Table_1.docx]

Supplemental Table 1: A summary of the number of transcripts (rows under the “tcts” heading), total number of bases (rows under the “bases” heading), N50 (rows under the “N50” heading), and BUSCO analysis (rows under the “BUSCO” heading) of the assembled transcriptome under various filtering and clustering parameters. The row titles correspond to the following filtering criteria; all ~ no filtering; all – FWP ~ all transcripts net of those with a hit to known fungal, *Wolbachia* or *Pantoea* genes; NR – WP ~ transcripts exhibiting a hit to a known NR database protein net of those with a hit to fungal, *Wolbachia* or *Pantoea* genes. Each column refers to different clustering criteria used by the cd-hit-est program (v4.8.1; Fu et al. 2012), where the unclustered column was not clustered and the subsequent columns were clustered using the same similarity threshold (i.e., the program’s ‘-c’ parameter) as noted in the column header (e.g., the 0.99 column was clustered with a sequence identity threshold of 0.99). BUSCO analysis was done using the hemiptera_odb12 (n=3396) dataset (Tegenfeldt et al. 2025) where the following abbreviations are used: C ~ complete, S ~ Single (only one copy of the given BUSCO gene was detected), D ~ Duplicated (at least two copies of the given BUSCO gene were detected), F ~ Fragmented (an incomplete copy of a given BUSCO gene was detected) and M ~ Missing (No evidence of a given BUSCO gene was detected).


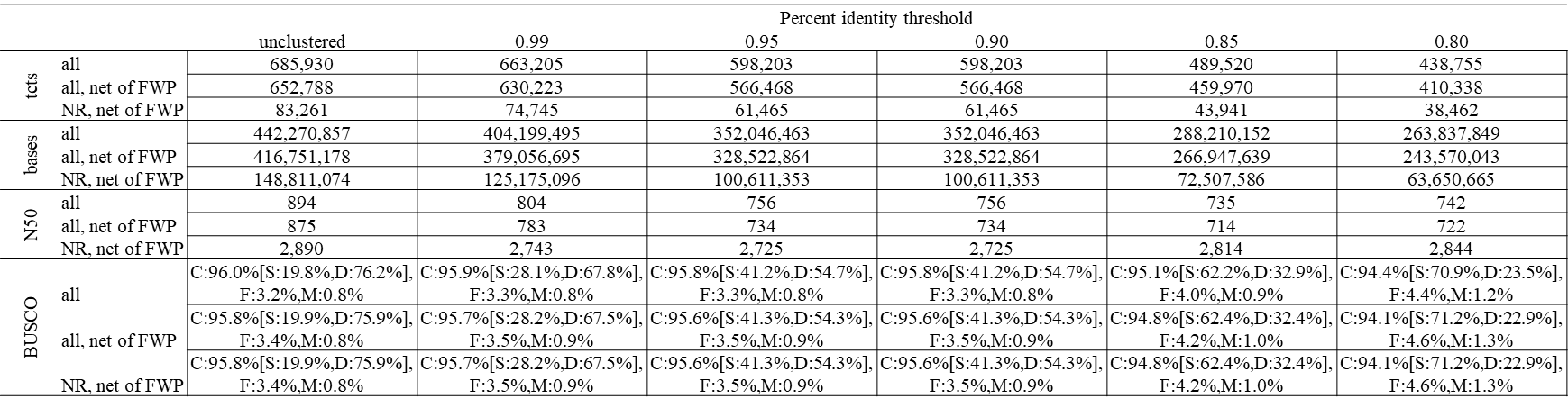


Note that clustering at the 0.95 and 0.90 percent identity thresholds yielded identical results. Note also that BUSCO results between the all-FWP and NR-FWP filtering levels are unchanged, because for these cases, all BUSCO genes considered here correspond to cotton seed bug transcripts that also exhibit a hit to sequences in the NR database. Clustering had an appreciable impact on the duplicated BUSCOs detected. This is likely because the global transcriptome assembly for this diploid organism was constructed from multiple individuals from a wild caught population, rather than an inbred lab culture, which can introduce a high degree of genetic variation. The high duplication rate seems due to multiple transcript variants of the same BUSCO belonging to genetically distinct individuals. Even at low levels of similarity (e.g., 80%) there is appreciable duplication, which would be expected of a transcriptomic dataset constructed from multiple samples occurring in a natural population.

Fu L., Niu B., Zhu Z., Wu S., Li W. (2012) CD-HIT: accelerated for clustering the next generation sequencing data. Bioinformatics, **28**:3150-3152

Tegenfeldt F., Kuznetsov D., Manni M., Berkeley M., Zdobnov E.M., Kriventseva E.V. (2025) OrthoDB and BUSCO update: annotation of orthologs with wider sampling of genomes. Nucleic Acids Research, **53**:D516–D522
